# Dr. Symonds on Therapeutics

**Published:** 1842-12-10

**Authors:** 


*Dr. Symonds on Therapeutics.*—The last and most important branch of medicine is therapeutics, the *ratio medendi.*

Medicine is the art of healing and relieving the sick, or it might otherwise be defined as the art of curing diseases; but, in using the latter terms, it is necessary to bear in mind the primitive sense of curing—that is, treating or taking the care or management of, there being many diseases, the cure of which, in the generally received sense of the word, is at present beyond our reach. That what I have hinted at is the original meaning, appears at once in the old saying, " Natura sanat, medicus curat morbos." I shall have frequent occasions hereafter to remind you that there is another difference between the two difinitions. It is one thing to cure diseases, and another to cure patients. The former may be done with great *éclat*, while the latter is left undone. To-day, by a vigorous use of the lancet and revulsive remedies, I may have put an end to an hæmoptysis; to-morrow my patient may die of syncope from the loss of blood.

I have said that medicine is an art; as such, it is an example of the subjugation of nature to the mind and will of man. It is not, as I shall presently attempt to show, a mere imitation of nature. Nature must be the instructress, yet only to become the servant of man. To master her powers he must observe them accurately; but when he has made them work his purposes, he cannot be said to have been the mere follower of nature, otherwise than as the workings of his own mind are a part of nature's operations, in which sense we lose sight of the common meaning of nature, which to the observer is objective. Art, then, is external nature moulded to the desires of man; just as nature in its widest signification, including man and the whole universe, has been sublimely said to be the " art of God."

The connections between art and science I shall not trace on the present occasion. I shall enter at once on the consideration of the principles on which the art of medicine is or ought to be practised.

Very different principles have been taught by different professors of the art. One of the oldest divisions among physicians was that of the empirical and the rational; the former professing to observe the action of remedies, and to give like remedies in like cases; the latter to ground the use of their measures on their knowledge of the functions of the body, the nature and causes of diseases, and the presumed fitness of remedies. The rational sect have also been called dogmatic or theoretical practitioners. Let us take an illustration. A patient is presented subject to epileptic fits; the empirical practitioner proposes to give him nitrate of silver, or indigo, or powdered misletoe, or some other one of the thousand specifics of vaunted efficacy in this disease. Ask him why he makes this suggestion. His answer, if he is a consistent empiric, will be, that he knows nothing of the modus agendi, but that from frequent observations on his own part, and that of others, he is satisfied that the disease is likely to disappear after the use of the nostrum; that is, he has seen it so removed once, twice, a dozen, or fifty times, and he expects a similar success in the case before him. But what says the rational practitioner? He begins with unfolding his view of the pathology of the case. He may consider, for instance, that during the paroxysms there is unquestionable evidence from the spasms, the convulsions, the loss of consciousness, that the vessels of the spinal chord and the brain are overloaded with blood; also that in the intervals the patient presents symptoms of tendency to congestion, though in less degree, of these organs; that the heart beats with undue impetus, and so forth; and therefore he opines that measures should be taken for relieving the distended blood-vessels during the fits, and for subduing the congestive tendency in the intervals, whether by reducing the amount of blood in the whole system, and lowering the action of the heart, or by equalising the circulation by derivants to the surface of the body and the extremities. Or he may take a somewhat different view. He may urge that though the paroxysmal symptoms certainly indicate a turgid state of the cerebro-spinal capillaries, yet this, like the morbid orgasm of other parts, may be owing far less to any general fault of the circulation in the way of excessive tone, than to extreme irritability of the nervous system which has engendered the morbid state of the capillary circulation; that to abstract blood, would be a sure way of aggravating the excitability, and that the most rational remedies would be such as soothe the nervous system when excited, and such as lessen its mobility, by increasing its tone; or he may lay down a third doctrine. He may say all this disturbance of the spinal marrow and brain is caused by some irritating agent operating upon the extremities of the incitor or afferent nerves, and that it is useless to direct measures against the nervous centres when the mischief lies in some part of the periphery. Let us investigate, says he, and we shall find some disturbing cause acting on the febrils of the fifth pair in the teeth, or on those of the par vagum in the stomach, or on the uterine nerves. Having found this, we must set to work and remove it, and then there will be no more eccentric irritation of the excito-motory system. To return, I suspect that the distinction between the above classes of practitioners, although it corresponds with the difference in the habits of thought common to two classes of men, has reference more to degree than to kind. There are few—very few—who are absolutely empirical. Under the guise of the most simple form of empiricism we can often detect a lurking theory. It is not in the nature of the human mind, however unphilosophical the individual may be, to observe facts at all times in simple sequence, without the intervention of some cause fancied by the individual. The most ignorant of our empirics not unfrequently in the very announcement of their pretended remedial discoveries, and while the very words of scoffing at theories and philosophers are yet on their tongues, betray the thraldom maintained over their minds by dogmas and hypotheses, which, though once dominant in the high places of philosophy, have been exiled in favor of other dynasties, and compelled to seek a more appropriate sway over the credulous vulgar. Or—to take a far more respectable instance of the truth of our remark—the savage who has seen the fever of his companion dissipated by drinking of a pool, in which a potent vegetable has accidently been macerated, does not content himself with the mere observation that his friend had a fever, that he drank of the water and recovered, but he views the fever as the production of a malignant spirit, and the water is to his apprehension endowed with some secret mysterious power "by the unseen genius of the wood."

Those who have been professedly rational practitioners have acted, as might well be expected, according to very different systems. Thus, we have the expectant method, the perturbant, the electic. One or two remarks may be sufficient to sketch their respective peculiarities.

The physicians of the expectant school are those who pin their faith to the "vis medicatrix naturæ,"—who think that if this power fails no other can be looked to for aid. They presume that in all cases curable at all, nature herself tends to bring about the desired result; and that the doctor has only to stand by and assist. He is to be emphatically the "interpres et minister naturæ," carrying out her designs; if her energies are languishing, he must endeavour to sustain them ; if they are too vehement, he may somewhat restrain them ; but always modestly and reverently. "Laissez faire" is ever the motto of this school. The favourite measures of the expectant physicians are—ptisans, emulsions, gentle alteratives, salines, placebos ; they lay great stress on diet and other hygienic agents, their chief object being to place the patient in circumstances as favourable as possible to the sanative operations of nature.

The opposite system is the medicina perturbatrix—a method of active interference, engendered by a want of confidence in the ability of nature to conduct the patient to a safe termination of his disorder, perhaps even by suspicions that her intentions even are by no means to be trusted; and that as often as not, she means to extinguish instead of fanning the flame of life. To a physician of this way of thinking, it seems that almost any change is better than the present state of disease, and he resorts to the most powerful artificial agents on the economy, preferring that the patient should die of the remedy rather than of the disease. With such practitioners, the lancet, blood-letting, evacuants, and revulsives in some cases, or powerful stimulants and narcotics in others, are the weapons most in use.

These are on either side extreme opinions, and not only extreme but exclusive, and as such erroneous. To look fixedly in one direction, and because truth is seen, to refuse to turn the eyes in another direction, and not only so, but to deny that truth can lie anywhere in the universe but on the very spot contemplated, is to err (if the phrase be not a solecism) from the want of discursiveness. Those who do not regard the processes of disease in so partial a manner, may readily discern two different tendencies, one to reparation and health, the other to destruction and death ; nay that the very same vital action which in one situation may be most salutary, in another may be just as pernicious. The effusion of fibrin, which, in an external wound, restores the continuity, may on the surface of the intestines produce fatal entanglements, on that of the heart may embarass the organ for the remainder of life, and in the air-passages become literally a fatal obstacle. The formation of pus in the liver may seem to have saved the patient, when the abscess has pointed through the parietes of the abdomen, or through one of the mucous outlets, but the same process is mortal when the matter escapes into the peritoneal cavity. There is strong reason for surmising that death was contemplated by the Divine Author of our being, as the result of our constitution no less than the continuance of life, and that disease is one of the manny means intended to accomplish the former of these purposes. If organic processes are so adjusted that they assist each other, that they recover from disorder immediately on the removal of the disturbing cause, and that fatal disease in one part is prevented by a morbid state supervening on another part; it is no less true that at other times these processes interfere with each other, that they persevere in morbid action, and that the occurrence of disease in a part of trivial importance will be the occasion of serious mischief in a vital organ ; that, in short, if on the one hand we observe harmony, reparation, and salutary revulsion, on the other we cannot shut our eyes to the existence of discord, destruction, fatal sympathy, and metastasis. Were we disciples of Zoroaster we might recognise in the human body, as throughout the universe, the sway of two antagonist principles, a good and an evil. The vis medicatrix is opposed by what I have been accustomed to call "vis vitiatrix." On contemplating these forces the practitioner may exclaim, "Non nostrum inter vos tantas componere lites."

But his duty is not that of an arbiter; he must be a steady partisan of the one—the deadly foe of the other; the difficulty is to be discreet in his aid; if he interferes rashly and unadvisedly he may thwart or even injure the very party he is anxious to abet. We have to study closely the natural course of maladies as to this very point; if it appears that the tendency to recovery is very marked, we should be foolish to interfere. If, on the other hand, it has been ascertained that cases left to themselves, or but little meddled with, more frequently end in death than in recovery, it is our plain duty to make trial of remedies, even though of the most doubtful description, on the old maxim, "anceps remedium melius quam nullum." In many cases of continued fever the practice may be expectant, in local inflammations it it can rarely be so with safety; yet in both instances, circumstances may arise to change the plan of treatment. In the fever a local disease may be set up requiring the most active exertions of the physicians, such as an intestinal hæmorrhage depending on follicular ulceration, and requiring the bold exhibition of some of our strongest medicines. Again, in the other case, the inflammatory process may be undergoing spontaneous cure by the increase of a natural secretion, and here the *nimia diligentia medici* might be as dangerous as inaction in the former instance. In brief, almost every case that comes under treatment requires the practitioner to be constantly on the watch, to determine not only how to interpose the aid of art, but when to do so.

In the practice of medicine my advice to you is to aim at being eclectic; do not be enticed into the notion that to be eclectic is to have no decided views, or, in other words, to be blowing hot and cold. If you care rather to save yourselves trouble than to do your duty towards your patients, you will find it unquestionably easier to take up with one exclusive system of pathology and apply to it one system of therapeutics. Individuals of great genius and vast knowledge have fallen into this error, as I shall have hereafter to point out to you; and when men have been the authors of useful discoveries we may make allowance for their having over-valued their own productions and fixed their minds too exclusively upon them; but we must not permit ourselves to be misled, and to adopt their errors, because it saves us the trouble of viewing other facts and doctrines. One cardinal maxim let me urge upon your adoption; suspect the truth of any therapeutical system of great simplicity, and the more so if propounded by one imperfectly acquainted with the fundamental departments of medical science; be certain that when anatomy and physiology are every day exhibiting more and more strongly the infinite complexity of the normal structures and functions, pathology and therapeutics must be proportionally composite. Take no account of what is called the overwhelming testimony to the efficacy of these simple methods when offered by uninformed observers. Such has been offered in favour of the most absurd and dangerous forms of quackery that have ever been propounded. If the testimony refer only to a single remedy for a single disease, or for a particular method for such disease, you may listen to and weigh it carefully; remembering that many remedies have in the first instance been purely empirical; but when the one remedy or plan of cure is to be applied to many and divers diseases, having little or no affinity with each other, you may safely dismiss it from your minds.

Let me, however, again recommend to you the eclectic method. Be not exclusive in your rejection of methods any more than in your adoption of them. How unwise would it be to forego the advantages of the stimulant treatment once so universally applied by the Brunonians, or of the bleeding of the Broussaists, or of the blue pill system of Abernethy, or of the "beef-steak and porter" practice of others, merely because the indiscriminate use of such measures has led to deplorable failures in practice. Each of these systems is good in the right place; "nullum remedium quin solo tempestivo usu tale fiat."

I would advise you even not to disdain taking a hint now and then from the practice of heretical professors, if you can turn it to account, for the good of your patients; for in most of the heresies in medicine, as in philosophy and religion, there may be detected a portion of truth, though overlaid by a mass of absurdity and error: to instance, two systems at the present time very popular, though one has passed its meridian of favour, and the other is in the ascendant—I mean homœopathy and hydropathy. The former of these has two grand principles—one, that diseases are cured by substances which produce such diseases, "similia similibus curantur," as bark is said to cure ague, because it produces ague in healthy persons, (though I confess that, in a pretty large experience of the use of this drug, I never saw any such effects;) or as diarrhœa is cured by purgatives, &c. The second principle is, that these substances must be given in infinitesimal doses, and that, in the trituration and agitation essential to such minute sub-divisions, new powers are developed. Now, to the first of these principles much exception may be taken, though there is an apparent plausibility in it. The artificial action is not the same, though somewhat like. The evacuations produced by the purgative that cures a diarrhœa are very different from those that constituted the latter, and argue a dissimilarity in the states of the membranes that furnished them. The inflammation produced in the eye by nitrate of silver is different in character from the inflammation which it is so useful in removing. Still something may be learned from the facts adduced in support of the dogma; and it is that, inasmuch as certain substances do produce a morbid change in certain organs, and that change is not only often subversive of spontaneous disease, but also itself easily removable, we may with benefit make use of such substances. Often the difficulty in practice is to find a substance that will act upon the part which we deem the seat of disease; but the discovery of one that induces an effect like the disease, shows that we have a substance likely to answer our purpose, of engendering in the seat of disease an artificial evil that may supersede that which is spontaneous. As to the other principle, I have no hesitation in saying that I utterly disbelieve it. No *a priori* evidence has been adduced in favour of it; and if my faith is to be obtained only by trying experimentally whether the decillionth of a grain of opium does or does not produce its reputed effects, I must remain a hardened infidel. To such egregious absurdity I say, "Incredulus odi." But what, you may ask, is to be made of the apparent homœopathic cures? Simply this, that the expectant or *laissez-faire* method is in this system carried out to the fullest extent. The natural tendencies to recovery have their full play, and are often aided at once by the suspension of meddling treatment, and by the judicious regulation of diet, the latter being all the positive operation of the homœopathic system, the rest only negative.

The fashionable hydropathy or hydriatics imported from Silesia consists in a vehement and universal employment of cold water, both internally and externally—the extended use of a remedy familiar to every well-informed practitioner. Perhaps something may be learned from this system as to the mode of applying the remedy; but one cannot help being amused with the pretensions of the hydropathic system, which is to overturn all other methods of cure, and to place the art of medicine on a new basis. This announcement would be startling did it emanate from persons acquainted with the structure and functions of the human body in health and disease; but issuing from persons devoid of this knowledge, we must receive it with a good-humoured smile, and regret that these sanguine ecomiasts have so much disappointment to encounter.

That cold water, in the form of the shower-bath, or cold sponging, or the plunge-bath, is an efficacious tonic, nine-tenths of the practitioners in this country are daily impressing on patients who require such means; and that it is an efficacious auxiliary in repressing certain inflammatory and hæmorrhagic diseases, is at the present moment experienced by the hundreds of sick persons who are ordered by their physicians to have cold cloths on their scalps in meningitis, to swallow iced drinks for cynanche, or gastritis, or hæmatemesis, or hæmoptysis, to have pails of cold water emptied over the abdomen for hæmorrhage after parturition, to dip into cold hip-baths for menorrhagia, or to keep their wounds wet with cold water dressings. If you have to learn how and when to give such directions you may find abundant instruction in the classical work of Dr. Currie, in the treatise on cold by M. Beaupré, in an admirable article on bathing by Dr. Forbes in the "Cyclopædia of Practical Medicine," and in many other practical works with which medical literature is adorned. Even from so old a work as that of "Mercurialis de Arte Gymnasticâ" you may glean much useful information respecting the application of cold water for hygienic purposes. It cannot be questioned that the mere ingestion and subsequent elimination of large quantities of liquids often produce most beneficial results; and their utility may be traced, as Dr. Holland has well shown, to "first, the mere mechanical effect of a quantity of liquid in diluting and washing away matters, excrementitious or noxious, from the alimentary canal; secondly, their influence in modifying certain morbid conditions of the blood; and thirdly, their effect upon various functions of secretion and excretion, and especially upon those of the kidneys and skin." (Med. Notes and Reflections, p. 316.) For obtaining knowledge of this kind, then, we have no need to depend on the inspirations of the heaven-born peasant-physician of Graeffenberg. In applying this epithet to Vincent Priessnitz, I have no wish to reflect upon his lowly origin, well-knowing how many lights of science have sprung from the humblest ranks of society; but it is proper to remind you that he who is proclaimed as the great reformer of medicine is still a peasant—an uncultured, uninstructed man, so far as medical knowledge is concerned.* I have reason, however, to believe that this individual is endowed with great natural sagacity, which is in no respect shown more remarkably than in his selection of favourable cases for the water cure; many invalids he rejects as unfit subjects for the process, and in so doing manifests a proper discernment. How many, alas! of the cases which daily require the anxious care of the ordinary medical practitioner must be rejected, were they capable of being transported to a water establishment!—*Provincial Medical Journal.* October 22, 1842.

* Mr. Claridge states that he doubts whether Priessnitz knows on which side of the body the liver is situated.

